# Microsatellite Instability in Colorectal Cancer Liquid Biopsy—Current Updates on Its Potential in Non-Invasive Detection, Prognosis and as a Predictive Marker

**DOI:** 10.3390/diagnostics11030544

**Published:** 2021-03-18

**Authors:** Francis Yew Fu Tieng, Nadiah Abu, Learn-Han Lee, Nurul-Syakima Ab Mutalib

**Affiliations:** 1 UKM Medical Molecular Biology Institute (UMBI), Universiti Kebangsaan Malaysia, Kuala Lumpur 56000, Malaysia; francistieng@yahoo.com.my (F.Y.F.T.); nadiah.abu@ppukm.ukm.edu.my (N.A.); 2 Novel Bacteria and Drug Discovery Research Group, Microbiome and Bioresource Research Strength, Jeffrey Cheah School of Medicine and Health Sciences, Monash University Malaysia, Selangor 47500, Malaysia; 3 Faculty of Health Sciences, Universiti Kebangsaan Malaysia, Kuala Lumpur 50300, Malaysia

**Keywords:** circulating tumor cells, circulating tumor DNA, cell-free DNA, microsatellite instability, colorectal cancer, non-invasive, liquid biopsy

## Abstract

Colorectal cancer (CRC) is the third most commonly-diagnosed cancer in the world and ranked second for cancer-related mortality in humans. Microsatellite instability (MSI) is an indicator for Lynch syndrome (LS), an inherited cancer predisposition, and a prognostic marker which predicts the response to immunotherapy. A recent trend in immunotherapy has transformed cancer treatment to provide medical alternatives that have not existed before. It is believed that MSI-high (MSI-H) CRC patients would benefit from immunotherapy due to their increased immune infiltration and higher neo-antigenic loads. MSI testing such as immunohistochemistry (IHC) and PCR MSI assay has historically been a tissue-based procedure that involves the testing of adequate tissue with a high concentration of cancer cells, in addition to the requirement for paired normal tissues. The invasive nature and specific prerequisite of such tests might hinder its application when surgery is not an option or when the tissues are insufficient. The application of next-generation sequencing, which is highly sensitive, in combination with liquid biopsy, therefore, presents an interesting possibility worth exploring. This review aimed to discuss the current body of evidence supporting the potential of liquid biopsy as a tool for MSI testing in CRC.

## 1. Introduction

Over the years, colorectal cancer (CRC) displayed a steady increase of incidence and mortality rates [1,2,3,4]. It was one of the top three causes of cancer besides ranking third for all cancer-related deaths in 2018 [1]. Despite the advancement in the management of resected CRC as well as the introduction of more effective cancer detection tools and treatment options, approximately 30 to 50% of the patients recovered will experience a recurrence in the form of regional lymph node or distant metastasis [5,6]. This data suggested the presence of potential metastatic cells, which had been overlooked by currently available diagnostic tools, limiting the identification of patients in need of adjuvant therapy.

The rare metastatic cells which released from primary or metastatic cancers into the blood circulation were reported as circulating tumor cells (CTCs) [7,8]. Previously, detection, isolation and molecular characterization of CTCs had been largely hindered due to their unknown frequency in metastatic CRC (mCRC) patients, low concentrations (one CTC per 10^7^ to 10^9^ hematological cells per mL) and technical limitations such as low separation efficiency and low recovery [9,10,11,12,13,14]. In recent years, advances in microfluidics and immunoaffinity enrichment technologies along with sequencing platforms, permit robust reproducible detection and isolation of CTCs from the whole blood, leading towards comprehensive interrogation of CTCs [10,15]. For instance, in 2017, Li et al., proposed a detection method using quantum dots and gold nanoparticles as signal probes [16], whereas Chiu et al., introduced an optically-induced-dielectrophoresis (ODEP)-based microfluidic device capable of isolating high-purity and integral CTC clusters in 2018 [17]. In response to the increasing popularity in CTCs, various studies were carried out to characterize CTCs based on their distinctive molecular and clinicopathological features [9,17,18,19].

The revolutionary success of checkpoint inhibitors in mismatch repair-deficient (MMR-D) mCRC [20] also resulted in a new therapeutic scenario, where biomarkers including microsatellite instability (MSI) status have been validated for clinical use of CRC [21,22,23,24,25]. Today, post-diagnosis MSI testing is recommended for both hereditary syndrome screening as well as prognosis and treatment implications in CRC patients [26,27]. In the future, MSI status obtained from CTCs could act as a guide in early detection, prediction and prognosis, as well as facilitating therapeutic target selection and monitoring CRC treatment response.

## 2. Pros and Cons of Current Tissue Biopsy-Based MSI Testing

**Figure 1:**
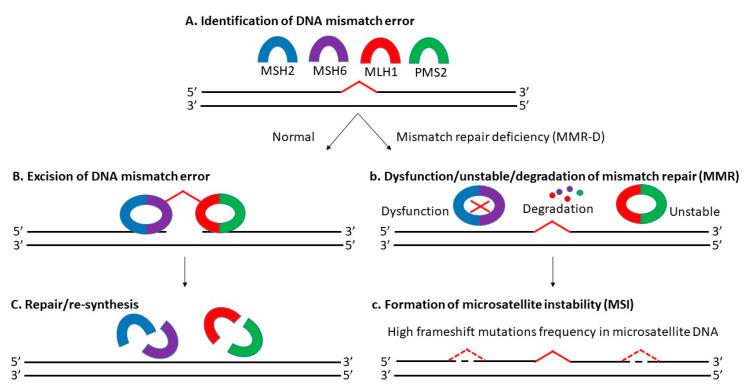
(**A**–**C**) Functioning mismatch repair system (MMR) capable of repairing DNA mismatch error, whereas (**b**,**c**) Defective mismatch repair system (MMR-D) which resulted in microsatellite instability (MSI).

Microsatellite instability (MSI) is the manifestation of defective mismatch repair (MMR), which results in high frameshift mutation frequency in microsatellite DNA, including gain and/or loss of nucleotides within repeating motifs known as microsatellite tracts (Figure 1) [28,29]. It is mainly dependent on MLH1 (MutL homolog 1), MSH2 (MutS homolog 2), MSH6 (MutS homolog 6), and PMS2 (postmeiotic segregation 2) proteins [30]. There are 3 types of MSI based on its frequency: high microsatellite instability (MSI-H), low microsatellite instability (MSI-L) and microsatellite stable (MSS) [31,32]. Approximately 12 to 20% of CRCs are explained by MSI-H due to MMR-D, with a higher incidence in the early stages (about 20% in stage I and II, and 12% in stage III) and a lower incidence in the metastatic setting (4 to 5%) [21,33,34]. Today, MSS-L and MSS are still classified as one kind, as there is no distinct phenotypes/genotypes linked with MSI-L determined by microsatellite markers [35,36].

Previously, several CRC classification systems with MSI status considered had been established including the Jass classification (2007) [37], Ogino classification (2008) [36], colon cancer subtyping (CCS) (2013) [38], colon cancer molecular subtyping (2013) [39] and CRC intrinsic subtyping (2014) [40]. Their attempt to improve CRC prognosis, diagnosis and targeted therapy based on the molecular subtyping alone was futile due to the unstandardized protocols, discrepancy in the number of subtypes, overlapping and mixed subtypes, and the lack of transcriptomic, genomic and proteomic data [41,42]. In 2015, Guinney et al., introduced the consensus molecular subtyping (CMS), where CRC was classified into 4 subtypes: (i) CMS1, MSI-H; (ii) CMS2 canonical, MSS; (iii) metabolite CMS3, MSI-L/moderate; and (iv) CMS4 mesenchymal [43]. They incorporated molecular subtyping with phenotypic signatures displayed by each subtypes to aid in disease stratification in routine pathology. Nevertheless, CMS was still limited due to the intratumoral heterogeneity (ITH) detected and unreliability in the tissue biopsy samples (43% unknown) [44,45]. In short, the lack of a standardized MSI-based classification system, the use of tissue biopsy samples, the presence of ITH and the variations of MSI status across different subtypes complicates CRC diagnosis and treatment.

Currently, MSI detection includes immunohistochemistry (IHC) and polymerase chain reaction (PCR) and is based majorly on tissue-biopsy samples [46,47,48,49]. Results from MSI detection is associated with improved prognosis and showed potential prognostic value especially during early stages of CRC [21,50]. For example, a data analysis from 17 separate trials in the Adjuvant Colon Cancer End Points (ACCENT) database had proven the potential of MSI status in predicting the outcomes/overall survival of patients with stage II or III CRC undergoing surgery with or without 5-fluorouracil (5-FU)-based adjuvant treatment [51]. Another previous subgroup analysis of the adjuvant QUASAR (Quick and Simple and Reliable) study verified the positive correlation between prognostic value and MSI status in early stages of CRC, where patients with MMR-D tumors had a higher recurrence rate (50%) than MMR-proficient tumors [52]. A recent study by Paulose et al., also highlighted the distinct clinicopathological features of MSI-related CRC and the relevance of MSI testing of stage II CRC for management decisions and prognostication [53].

Despite all the advantages and being recommended by clinical practice guidelines (NCCN), tissue biopsy-based MSI detection was not incorporated into the routine analysis, due to its inherent invasive nature of traditional biopsies; inability to interrogate full tumor load’s heterogeneity [54]; difficulties in repeated sampling; lack of viable tissue and not being routinely available; unavailability/infeasibility in certain patients [55]; and requirement of an adequate amount of tissue with a high concentration of cancer cells (paired with normal tissues) [56,57]. Detection of MSI by PCR via fragment analysis was not ideal in the clinic since it required samples of both tumor and normal tissue. Furthermore, PCR-based procedure was complex and involved additional specialized equipment, while being low sensitive for samples with low proportions of cancer cells [58]. Conversely, IHC-based MSI detection involved interpretations from pathologists, which could be subjective and highly susceptible to technical factors [57,59]. To sum up, the limitations of currently available tissue-based MSI detection offsets the prognostic and therapeutic intervention (prediction) values of MSI status in CRC, urging the necessity of a more precise and rapid non-invasive detection of MSI status in clinical routine analysis for better survival outcomes among CRC patients [20,60,61].

## 3. Necessity of Liquid Biopsy Specimens in MSI Testing

Liquid biopsy is a minimally invasive technique for the detection of prognostic or diagnostic tumor-derived markers in body fluids [62]. Previously, studies had proven implementation of biomarkers from blood as a non-invasive method for CRC screening, particularly during its early stages (stage I or premalignant stage) [63,64,65,66,67]. Examples of the markers evaluated were (1) proteins (hemoglobin) [68,69,70,71]; (2) deoxyribonucleic acid/DNA from intact cells or blood circulation (including methylation markers) [72,73,74,75,76,77]; (3) ribonucleic acid/RNA (messenger RNA, non-coding RNA and microRNA) [78,79,80,81]; (4) genes (mutation) [82,83]; and (5) low molecular weight metabolites (volatile organic compounds) [84]. Since the approval of first liquid biopsy-based test by the Food and Drug Administration (FDA) in 2016, numerous blood-based detection methods have been the focus of CRC screening to overcome the above-mentioned difficulties in traditional tissue biopsy testing [85,86,87].

Benefits of these liquid biopsy-based over conventional tissue-based biopsies MSI testing includes rapid detection; non-invasive procedures; high concordance rate with tissue biopsy-based detection; high specificity, precision, and sensitivity; ability to monitor genetic heterogeneity; and potential to enhance utility of tumor detection assays to help direct clinicians beyond targeted therapies to include immunotherapies [60,88,89,90,91]. Although introduction of next-generation sequencing (NGS) and computational algorithms enables unbiased, genome-wide screening of the molecular fingerprints of MSI with increased sensitivity, liquid biopsy-based MSI detection still appears to be in early development due to great technical and bioinformatics challenges (in efficient molecular capture, sequencing, mapping, variant calling, error correction at MSI loci); low tumor fraction in circulation; and high level of technical noise due to polymerase slippage [73,92,93]. In short, more advancement is required before liquid biopsy-based MSI detection could be incorporated into the routine analysis.

**Figure 2:**
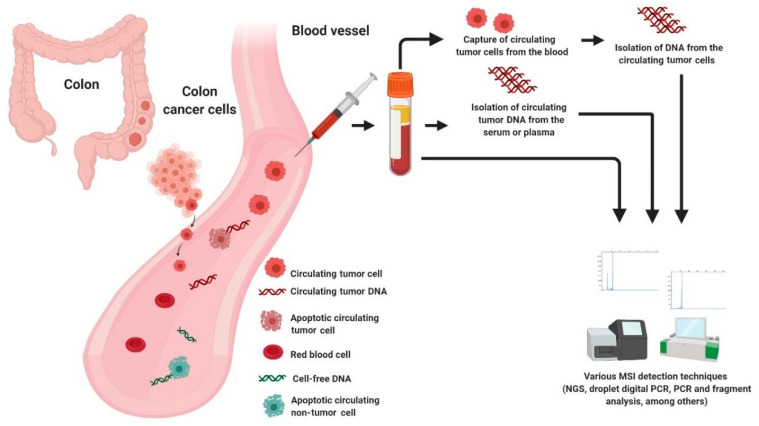
Possible sources of DNA for MSI testing in liquid biopsy.

One key element from both academic and commercial interest is circulating cell-free DNA (cfDNA) [94]. According to literature, cfDNA referred to fragmented DNA in the bloodstream from the secretion of necrotic or apoptotic cells and active release by intact cells. It comprised of both tumor-derived DNA (also known as circulating tumor DNA or ctDNA) and DNA from non-tumor origins, including hematopoietic, immune and blood stromal cells [95,96,97]. The possible sources of cfDNA are illustrated in Figure 2. Additionally, ctDNA is defined by mutations and genomic changes that are hallmarks of cancer and is a potential surrogate for the entire tumor [98].

### 3.1. Cell Free DNA (cfDNA) and Circulating Tumor DNA (ctDNA)

Although there were several proposed screening approaches using cfDNA and ctDNA, their feasibility in routine screening, given biological, technical and practical considerations were questioned [99,100]. While this may be true, they are still extensively studied, especially in MSI detection due to the minimally-invasive procedure with high specificity and ability to address genetic heterogeneity and capture the mutational landscape of CRC patients (Table 1) [101,102,103,104]. To illustrate this, in 2017, Kasi identified a threshold for recognizing MMR-D and MMR-proficient tumors. The power of cfDNA testing in capturing the tumor mutational burden (TMB) from two CRC MMR-D patients elucidated the potential of cfDNA as a surrogate marker for MMR-D or MSI [105].

In the subsequent year, Barzi and coworkers developed a cfDNA-based MSI testing by comparing microsatellite alterations in cfDNA with those in genomic DNA extracted from buffy coats from the same patient. Not only did the analysis differentiate MSI-H and MSS tumors effectively, but it also reported a distinct immunotherapy response based on the different tumor status of MSI [106]. In 2019, Georgiadis et al., invented a hybrid-capture-based 98-kb pan-cancer gene panel with a multifactorial error correction method and a novel peak-finding algorithm, capable of identifying rare MSI frameshift alleles in cfDNA. They demonstrated the feasibility of this non-invasive pan-cancer screening in capturing the targeted MSI locus and predicting the progression-free survival of MSI or tumor mutation burden-high patients treated with PD-1 blockade [107,108]. In the same year, Isaacs et al. unraveled that detection of MSI status from cfDNA was possible via the Guardant Health Omni 2.0 mb panel. MSI-H cancer patients resistant to immune checkpoint blockade showed RNF43, APC and/or CTNNB1 mutations, suggesting the importance of co-activation of the WNT/B-Catenin pathway [109].

On the other hand, droplet digital PCR (ddPCR) is an improved PCR technology commercially available almost a decade ago [110]. This technology uses Taq polymerase in a typical PCR reaction to multiply the target DNA fragment from a complex sample using primer or primer/probe assays. Before amplification, the PCR reaction is partitioned into thousands of reaction droplets, and data acquisition occurs at the end point [111]. When this modification is implemented in the MSI assay, the analytical sensitivity of MSI-ddPCR has increased by at least 2 times higher than the detection threshold of the gold standard pentaplex test [112,113]. The feasibility of MSI-ddPCR has been explored largely in the tissues, and recently gains attention for its application in liquid biopsy [89]. In 2020, Silveira et al., demonstrated that MSI-ddPCR assays detected MSI in blood samples from all the patients tested (cfDNA), yielding a clinical specificity and accuracy of 100% [89].

To date, there were only two studies involving the MSI detection in ctDNA [114]. For instance, a group of scientists from China reclassified 13 CRCs based on their MSI status from ctDNA by amplicon-based NGS. Their results were also in concordance with those of tumor tissue origin. In other words, NGS-based testing could detect MSI status precisely from both blood plasma and tumor tissues with 100% sensitivity, unlike conventional PCR-based MSI assay. In short, their findings gave critical insights towards the potential of MSI from ctDNA as non-invasive prognostic and diagnostic markers among CRC patients [61].

In 2019, Willis et al., performed ctDNA testing using the Guardant360 NGS kit (Guardant Health Clinical Laboratory, Redwood City, CA, USA) combining 99 putative microsatellite loci and precisely identified 87% (71/82) of tissue MSI-H and 99.5% of tissue microsatellite stability (863/867) with an 98.4% overall accuracy (934/949). Not only was this ctDNA-based MSI detection (from plasma) highly concordant with tissue-based MSI testing, it also showed higher specificity, precision, and sensitivity, with a limit of detection of 0.1% tumor content [60,86].

### 3.2. Circulating Tumor Cell (CTC)

Recently, CTCs have emerged as a new spotlight in MSI research as they permit structural evaluation and molecular characterization of cancer phenotype as well as a snapshot of tumor heterogeneity in CRC [115,116,117,118]. In 2020, Toh et al., reveal the relationship of MSI with the increase in intra-operative and post-operative release of CTCs [119]. Unlike ctDNA, the intact CTCs consist of a heterogeneous pool of tumor cells with potentially resistant clones. In other words, CTCs contain additional DNA information which could possibly scan all MSI loci when compared to cfDNA [120]. Moreover, advancement in microfluidics and immunolabeling techniques allow reliable and specific isolation of CTCs [15]. Due to this, numerous efforts had been carried out to determine the validity of harnessing CTCs for MSI typing. To prove this, a comprehensive genomic study of CRC in 2014 identified heterogeneity in MSI and mutations in key genes such as *KRAS* and *TP53*, between tissue and CTC and between individual CTC. Interestingly, MSI was detected in some of the individual CTCs but not in the tumor, but none showed proof with prognostic impact [121].

In another attempt, Kong and colleagues have found that somatic mutations detected in CTCs are associated with CRC prognosis, and their mutation signatures are similar to tumor signatures. Notably, their findings also reinforced the clinical utility of CTC analysis beyond the prediction of disease outcome based on CTC count. However, they did not prove the link between mutation frequency and MSI status due to the small sample size [122]. Following this, in 2019, Messaritakis et al., proved that CEACAM5mRNA-positive CTCs from blood was an adverse prognostic factor (shorter overall survival) correlated with poor clinical outcome in mCRC patients with MSI-high tumors [123]. In short, although existing studies did not show any strong scientific evidence on the direct link between MSI from CTCs and their prognostic impact among CRC patients, their findings gave novel insights towards the application of CTCs in MSI setting. Table 1 summarizes the recent findings of MSI in cfDNA, ctDNA and CTCs from CRC.

## 4. Patents in Liquid Biopsy-Based MSI Test

A recent patent search conducted on 3 March 2021 on Lens.org resulted in only 10 patents since 2018 (https://link.lens.org/p57zyqkfXpg). Despite the supremacy of CTCs over fresh tissue, formalin-fixed paraffin-embedded (FFPE), cfDNA and ctDNA samples in obtaining sufficient high-quality DNA, no patents are being applied or granted to prove their exact roles in MSI detection. There are, however, several patents involving cfDNA and ctDNA as source materials. For instance, in 2018, Georgiadis and Sausen invented a method for MSI detection in a cancer patient via liquid biopsy with sample preparation using hybrid capture and non-unique barcodes [125]. In their claim, cfDNA fragments from blood or plasma of a patient were isolated to obtain sequences of a plurality of tracts of nucleotide repeats. Non-unique barcodes were attached to these fragments for identification of a group of sequence reads and their length. Amplification of at least one MSI locus (*NR-21*, *BAT-25*, *BAT-26*, *NR-24*, *MON0-27*, *Penta C*, and *Penta D*) was carried out. A report describing the MSI status was generated by determining distribution of lengths of the plurality of tracts that had peaks that deviated significantly from peaks in a reference distribution (matched normal DNA). This patent included four categories of claims, which were C07K16/30 immunoglobulins from tumor cells, C12N15/11 DNA or RNA fragment-modified forms thereof DNA or RNA not used in recombinant technology, C40B40/06 libraries containing salts of organic compounds classified in the groups for the libraries containing the parent compounds and G06F19/22 electric digital data processing for sequence comparison involving nucleotides or amino acids, e.g., homology search, motif or single-nucleotide polymorphism (SNP) discovery or sequence alignment.

In the same year, Huang from NantOmics company also filed a patent for MSI detection in a solid tumor without the need of tumor tissue [126]. Briefly, cell-depleted fraction or tumor DNA (ctDNA from serum) and cell-containing fraction or matched normal DNA (nuclear DNA from leukocytes) were isolated as starting material, followed by PCR amplification of at least one MSI loci (*NR-21*, *BAT-25*, *BAT-26*, *NR-24*, and *MONO-27*). The size difference was then performed using capillary electrophoresis, polyacrylamide gel electrophoresis, mass spectroscopy, chip-based microfluidic electrophoresis, and denaturing high-performance liquid chromatography without fluorescent markers. MSI status was determined based on the comparison of peak shape and position in an elution profile of a chromatogram of the amplified MSI locus via a step of independent component analysis. This group was granted a patent under three categories of claims, which were C12Q1/6886 nucleic acid products used in the analysis of nucleic acids; C12Q2600/156 polymorphic or mutational markers; and C12Q1/6827 for detection of mutation or polymorphism.

More recently, Rabizadeh disclosed a protocol, whereby identification of molecular alterations associated with MSI from cell-free nucleic acid (cfNA) derived from the blood sample of patients with or suspected MMR deficient cancer, resulted in treatment responsiveness and outcome prediction of treatment regimen comprising at least one checkpoint inhibitor [127]. Granted in 2019, this patent covered three categories, which were C07K16/30 immunoglobulins from tumor cells; C12Q1/6827 for detection of mutation or polymorphism; and C12N15/11 DNA or RNA fragment-modified forms thereof DNA or RNA not used in recombinant technology.

## 5. Clinical Trials for the Detection of MSI in Circulating Tumor DNA (ctDNA)

Clinical trials assessing the feasibility of MSI detection using liquid biopsy are currently ongoing (NCT03594448 [128] and NCT03561350 [129]). The University of Southern California in collaboration with the National Cancer Institute (NCI) is actively recruiting participants for the clinical trial to detect MSI in the ctDNA of stage IV CRC patients [128]. This observational prospective trial aims to determine the concordance between the electrophoretic mobility profiles of microsatellite biomarkers in circulating cell-free deoxyribonucleic acid (ccfDNA) versus primary tumor tissues in CRC patients exhibiting MSI. The trial also seeks to establish the link between changes in the electrophoretic mobility profile of microsatellite biomarkers in liquid biopsies from CRC patients with therapeutic responsiveness measured based on Response Evaluation Criteria in Solid Tumors (RECIST) criteria. Finally, the trial seeks to determine whether the microsatellite alleles (produced from liquid biopsy-based MSI testing of CRC patients) epitomize the whole cancer cell population or only a subgroup of cancer cells differentially affected by genomic instability.

Another ongoing trial by Shanghai Minimally Invasive Surgery Center in Ruijin Hospital is also actively recruiting participants for a trial to determine MSI status in the blood sample of advanced CRC patients by NGS [129]. In this trial, the ctDNA and leucocyte will be extracted from the blood sample for MSI detection by ColonCore NGS panel. If the result is positive, the MSI status could be easily learned without the acquisition of tissue samples.

In the United States, 308 participants were recruited for an intervention study (MK-3475-177/KEYNOTE-177) [130]. This ongoing phase III clinical trial aims to compare an immunotherapy drug (pembrolizumab) with chemotherapy in the stage IV CRC treatment. Briefly, the participants displaying MSI-H or MMR-D advanced CRC will be randomly assigned to receive either pembrolizumab or the investigator’s choice of 1 of 6 standard-of-care chemotherapy regimens (mFOLFOX6; mFOLFOX6 and bevacizumab; mFOLFOX6 and cetuximab; FOLFIRI; FOLFIRI and bevacizumab; FOLFIRI and cetuximab). The investigators hypothesized that pembrolizumab would extend progression-free survival relative to the current standard of care chemotherapy. Its previous phase II trial results from the KEYNOTE-164 trial had shown that patients with previously-treated MSI-H MMR-D metastatic CRC who received pembrolizumab responded well to the drug [131].

## 6. Challenges and Future Directions

To sum up, liquid biopsy-based MSI detection has emerged as the focus of future research on precision diagnosis and treatment of tumors [132]. It shows potential as a mass screening tool for early CRC diagnosis due to its non-invasive nature and supersedes traditional colonoscopy and immunochemical fecal occult blood test (iFOBT) with minimal risk of perforation, higher sensitivity, rapid simple procedure (blood draw) and without any pre-requirement (dietary restriction and extensive bowel preparation) [72,133,134,135,136,137]. However, there are still crucial remaining challenges to their wider use and implementation to clinical settings (Table 2) [138]. First and foremost, liquid biopsy-based MSI detection is limited in low or non-shedding tumors. Sample collection from CRC patients of early stages, low shredding rates of metastases, tumor heterogeneity and genomic subtype, presence of certain tumor mutations and having undergone cancer treatment, resulted in harvesting of low frequency of analytes (cfDNA or CTCs) [139,140,141]. Second, there is a lack of standard procedures for the sample preparation or the pre-analytical phase for liquid biopsy, resulting in difficulties to compare obtained results across different methodology approaches [142,143,144,145]. Third, the diagnostic accuracy and sensitivity of current available liquid biopsy assays are limited [99,146]. Fourth, sophisticated multicenter clinical validation studies and regulatory guidelines are lacking but must be established to ensure future clinical utility [147]. For instance, sensitivity at low allele frequencies and sequencing of MMR genes are still limited [148].

Fifth, liquid biopsy-based MSI testing is difficult when applying in localized cancer (low TMB) because the detection rate of mutations from liquid biopsy samples is relatively low. In particular, accurate tumor information can only be obtained when the abundance of ctDNA is greater than or equal to 10% [149]. Furthermore, highly sensitive techniques like digital PCR (dPCR) and ddPCR are limited to a very small number of genomic loci, and the specific mutation to be assayed is typically determined a priori. Sixth, even if the profiling of larger genomic regions is possible with NGS technique, their potential diagnostic utility is still hindered due to the low signal-to-noise ratio (presence of contaminating non-tumor cells such as hematopoietic cells, immune cells and blood stromal cells) [150]. Seventh, although the introduction of NGS-based MSI testing enabled evaluation of hundreds or even thousands of MSI loci and do not require a tumor-normal comparison [151], it presents challenges at low levels of MSI due to polymerase slippage (‘stutter’) that generate high false-positive rates at positions of homopolymers [91]. Other factors causing the sequencing artifacts errors are DNA damage introduced during sample storing, fixation and/or extraction steps; PCR errors/chimeras due to shearing in sequencing library preparation; base incorporation errors; incorrect alignment to the genome; and imperfect imaging during sequence data acquisition [152,153,154].

Last but not least, current available liquid biopsy-based MSI assays lack the ability to dissect ITH in CRC efficiently, hence we believe single-cell approaches are vital for MSI detection [155]. To prove this, a group of researchers from Beijing developed a robust method for the analysis of single cellular genomic mutations in 2017, capable of detecting MSI of every single cell within the intestinal metaplasia [156]. Thus, together with the rapid advancement in single-cell isolation technology, there is a promising potential of using single CTC to evaluate the MSI status in CRC via its ctDNA [157,158]. In short, a better understanding of the underlying mechanisms involved in the release of liquid biopsy components and adoption of comprehensive multidimensional profiling strategies are fundamental in solving these challenges.

## 7. Conclusions

Since CRC is one of the most prevalent cancers in humans and causes a remarkable public health problem worldwide, identifying the ways of diagnosis and treatment of CRC is of the most importance. MSI is an important marker in CRC which could aid diagnosis and prognosis as well as predicting the efficacy of chemotherapeutic and immunotherapy treatments. Over the years, advances in NGS technologies and computational algorithms have permitted impartial, genome-wide screening of MSI fingerprints to dramatically increase the sensitivity of MSI detection. Advancement in the capture of ctDNA and cfDNA is also remarkable. Sensitive MSI detection in liquid biopsies, however, still seems to be in early development. Nevertheless, a liquid biopsy-based test to evaluate MSI may hit a wider subset of patients, including those with insufficient tissue or when safety concerns about invasive surgery arise.

## Figures and Tables

**Table 1:** Summary of recent findings of MSI in cfDNA, ctDNA and CTCs from CRC.

Source	Type	Year	Finding	Citation
cfDNA	2 patients with MMR-D CRC tumors	2017	Effectiveness of the TMB report from cfDNA to predict MMR-D or MSI status and the possible response towards immunotherapy among CRC patients.	[105]
cfDNA	13 CRC patients	2018	Feasibility of MSI detection in cfDNA with possible reflective of TMB in CRC. MSI assessment from cfDNA could predict clinical outcomes of immunotherapy.	[106]
cfDNA	Plasma from 29 metastatic cancers (19 CRC, 3 ampullary, 3 small intestine, 2 endometrial, 1 gastric, and 1 thyroid cancer)	2019	Development of a hybrid-capture-based 98 kb pan-cancer gene panel with a multifactorial error correction method and a novel peak-finding algorithm, capable of predicting progression-free survival in MSI and TMB-High patients treated with PD-1 blockade.	[107]
cfDNA	1145 archived samples (residual plasma and/or cfDNA) collected and processed as part of routine standard-of-care clinical testing in the Guardant Health CLIA laboratory	2019	MSI assessment from cfDNA showed higher specificity, accuracy and sensitivity, with a detection limit of 0.1 percent of the tumor content than conventional tissue biopsy-based MSI detection.	[60]
cfDNA	Blood samples from 12 patients with MSI-H gastrointestinal tract cancer	2019	Detection of MSI status from cfDNA was possible via the Guardant Health Omni 2.0 mb panel. MSI-H cancer patients resistant to immune checkpoint blockade showed RNF43, APC and/or CTNNB1 mutations, suggesting the importance of co-activation of the WNT/B-Catenin pathway.	[109]
cfDNA	30 plasma or serum from 14 patients with locally advanced CRC, mCRC or endometrial tumors	2020	The MSI-ddPCR assays were clinically sensitive, highly accurate and appropriate for the quantitative ctDNA detection in observational studies.	[89]
cfDNA	cfDNA sequencing data from 39 patients and 1565 WES samples from TCGA were treated as the training set	2021	Development of MSIsensor-ct, a bioinformatics tool based on a machine learning protocol, dedicated to detect MSI status using cfDNA sequencing data with 100% accuracy within the LOD of 0.05% ctDNA content.	[124]
ctDNA	Plasma, matched tumor tissue and blood samples from 200 patients	2018	Correct identification of 13 MSI-H patients by MSI testing in ctDNA, in concordance with the results of MSI testing in tumor tissue with a sensitivity of 100%.	[61]
ctDNA	Plasma isolated from the peripheral blood from 222 consecutiveEGFR, KRAS, BRAF, and/or ESR1-positive NSCLC, colorectalcancer, or breast cancer patients	2019	Cell-free circulating tumor DNA-based MSI detection using Guardant360 was highly concordant with tissue-based testing, enabling highly accurate detection of MSI status concurrent with comprehensive genomic profiling.	[86]
CTCs	8 single CTC from 8 individual CRC patients with matched tumor tissue	2014	Identification of disparity in MSI status between primary tumor, liver metastasis and individual isolated CTC.	[121]
CTCs	CTCs from peripheral blood of 198 mCRC patients	2019	Detection of CEACAM5 mRNA-positive CTCs as an adverse prognostic factor which correlated with poor clinical outcomes in MSI-high tumors patients.	[123]

cfDNA = cell-free DNA, CRC = colorectal cancer, ctDNA = circulating tumor DNA, CTC = circulating tumor cell, ddPCR = droplet digital polymerase chain reaction, LOD = limit of detection, MMR-D = mismatch repair deficiency, mCRC = metastatic CRC, MSI = microsatellite instability, NSCLC = non-small cell lung cancer, TMB = tumor mutation burden, WES = whole exome sequencing.

**Table 2:** Summary of pros and cons of MSI testing in tissue and liquid biopsies.

Type	Advantages	Disadvantages
Tissue biopsy	Clinically validated; Gold standard for MSI detection (IHC and PCR); Potential CRC prognosis based on MSI status (especially during early stages of CRC); Allow prediction of the outcomes/overall survival of MSI-related CRC patients; Potential management decisions and prognostication; Allow distinct clinicopathological features of MSI-related CRC.	Not incorporated into the routine analysis; Invasive with high risk; Inability to interrogate full tumor load’s heterogeneity; Difficulties in repeated sampling; Lack of viable tissue and not routinely available; Unavailability/infeasibility in certain patients; Requirement of an adequate amount of tissue with a high concentration of cancer cells (paired with normal tissues) for PCR-based MSI detection; The need of specialized laboratory equipment (PCR) and professional expertise (e.g., pathologist) (IHC); Low sensitive for samples with low proportions of cancer cells; Impractical for periodic/real-time monitoring of cancer progression and treatment response.
Liquid biopsy	Rapid detection; High specificity; Non-invasive procedures and minimal risk; High concordance rate with tissue biopsy-based detection; Potential to monitor genetic heterogeneity; Ability to capture the mutational landscape of CRC patients; Capable of capturing TMB; Identification of rare MSI frameshift alleles; High repeatability and easily reproducible; Potential to enhance utility of tumor detection assays to help direct clinicians beyond targeted therapies to include immunotherapies; Possible to perform continuous follow up examinations.	Still in early development without established clinical practice rules; Lack of sophisticated multicenter clinical validation studies and regulatory guidelines; Unstandardized laboratory procedures; Limited diagnostic accuracy and sensitivity; Low detection rate of mutations; Inability to reflect ITH; Great technical and bioinformatics challenges (inefficient molecular capture, sequencing, mapping, variant calling, error correction at MSI loci); Low tumor fraction in circulation; High level of technical noise due to polymerase slippage; Low signal-to-noise ratio (presence of contaminating non-tumor cells such as hematopoietic cells, immune cells and blood stromal cells); Risk of false-positive and false-negative results; Microenvironment changes may influence the release or the amount of biological materials.

CRC = colorectal cancer, IHC = immunohistochemistry, ITH = intratumoral heterogeneity, MSI = microsatellite instability, PCR = polymerase chain reaction, TMB = tumor mutation burden.

## Abbreviations

ACCENT	Adjuvant Colon Cancer End Points
CRC	Colorectal cancer
cfDNA	Cell-free DNA
ccfDNA	Circulating cfDNA
CCS	Colon cancer subtyping
cfNA	Cell-free nucleic acid
CMS	Consensus molecular subtyping
CTC	Circulating tumor cell
ctDNA	Circulating tumor DNA
dPCR	Digital PCR
ddPCR	Droplet dPCR
DNA	Deoxyribonucleic acid
FDA	Food and drug administration
FFPE	Formalin-fixed paraffin-embedded
GI	Genomic instability
iFOBT	Immunochemical fecal occult blood test
IHC	Immunohistochemistry
ITH	Intratumoral heterogeneity
LS	Lynch syndrome
mCRC	Metastatic CRC
MLH	MutL homolog
MMR	Mismatch repair
MMR-D	Mismatch deficiency
MSH	MutS homolog
MSI	Microsatellite instability
MSI-H	MSI-high
MSI-L	MSI-low
MSS	Microsatellite stable
NGS	Next-generation sequencing
PCR	Polymerase chain reaction
PMS	Postmeiotic segregation
RNA	Ribonucleic acid
TMB	Tumor mutation burden.

## References

[1] Siegel R.L., Miller K.D., Jemal A. (2020). Cancer statistics, 2020. CA Cancer J. Clin..

[2] Arnold M., Sierra M.S., Laversanne M., Soerjomataram I., Jemal A., Bray F. (2017). Global patterns and trends in colorectal cancer incidence and mortality. Gut.

[3] Granados-Romero J.J., Valderrama-Treviño A.I., Contreras-Flores E.H., Barrera-Mera B., Herrera Enríquez M., Uriarte-Ruíz K., Ceballos-Villalba J.C., Estrada-Mata A.G., Alvarado Rodríguez C., Arauz-Peña G. (2017). Colorectal cancer: A review. Int. J. Res. Med. Sci..

[4] Bray F., Ferlay J., Soerjomataram I., Siegel R.L., Torre L.A., Jemal A. (2018). Global cancer statistics 2018: GLOBOCAN estimates of incidence and mortality worldwide for 36 cancers in 185 countries. CA Cancer J. Clin..

[5] Arvelo F. (2015). Biology of colorectal cancer. Cancer.

[6] Engstrand J., Nilsson H., Strömberg C., Jonas E., Freedman J. (2018). Colorectal cancer liver metastases—A population-based study on incidence, management and survival. BMC Cancer.

[7] Ashworth T.R. (1869). A Case of Cancer in Which Cells Similar to Those in the Tumors Were Seen in the Blood after Death. Australas. Med. J..

[8] Ferreira M.M., Ramani V.C., Jeffrey S.S. (2016). Circulating tumor cell technologies. Mol. Oncol..

[9] Hayes D.F., Smerage J.B. (2010). Circulating Tumor Cells. Progress in Molecular Biology and Translational Science.

[10] Krebs M.G., Hou J.-M., Ward T.H., Blackhall F.H., Dive C. (2010). Circulating tumour cells: Their utility in cancer management and predicting outcomes. Ther. Adv. Med. Oncol..

[11] Miller M.C., Doyle G.V., Terstappen L.W.M.M. (2010). Significance of Circulating Tumor Cells Detected by the CellSearch System in Patients with Metastatic Breast Colorectal and Prostate Cancer. J. Oncol..

[12] Andree K.C., van Dalum G., Terstappen L.W.M.M. (2016). Challenges in circulating tumor cell detection by the CellSearch system. Mol. Oncol..

[13] Van der Toom E.E., Verdone J.E., Gorin M.A., Pienta K.J. (2016). Technical challenges in the isolation and analysis of circulating tumor cells. Oncotarget.

[14] Zou D., Cui D. (2018). Advances in isolation and detection of circulating tumor cells based on microfluidics. Cancer Biol. Med..

[15] Bankó P., Lee S.Y., Nagygyörgy V., Zrínyi M., Chae C.H., Cho D.H., Telekes A. (2019). Technologies for circulating tumor cell separation from whole blood. J. Hematol. Oncol..

[16] Li X., Chen B., He M., Wang H., Xiao G., Yang B., Hu B. (2017). Simultaneous detection of MCF-7 and HepG2 cells in blood by ICP-MS with gold nanoparticles and quantum dots as elemental tags. Biosens. Bioelectron..

[17] Chiu T.-K., Chao A.-C., Chou W.-P., Liao C.-J., Wang H.-M., Chang J.-H., Chen P.-H., Wu M.-H. (2018). Optically-induced-dielectrophoresis (ODEP)-based cell manipulation in a microfluidic system for high-purity isolation of integral circulating tumor cell (CTC) clusters based on their size characteristics. Sens. Actuators B Chem..

[18] Barbazán J., Alonso-Alconada L., Muinelo-Romay L., Vieito M., Abalo A., Alonso-Nocelo M., Candamio S., Gallardo E., Fernández B., Abdulkader I. (2012). Molecular Characterization of Circulating Tumor Cells in Human Metastatic Colorectal Cancer. PLoS ONE.

[19] Dizdar L., Fluegen G., van Dalum G., Honisch E., Neves R.P., Niederacher D., Neubauer H., Fehm T., Rehders A., Krieg A. (2019). Detection of circulating tumor cells in colorectal cancer patients using the GILUPI CellCollector: Results from a prospective, single-center study. Mol. Oncol..

[20] Le D.T., Uram J.N., Wang H., Bartlett B.R., Kemberling H., Eyring A.D., Skora A.D., Luber B.S., Azad N.S., Laheru D. (2015). PD-1 Blockade in Tumors with Mismatch-Repair Deficiency. N. Engl. J. Med..

[21] Battaglin F., Naseem M., Lenz H.-J., Salem M.E. (2018). Microsatellite Instability in Colorectal Cancer: Overview of Its Clinical Significance and Novel Perspectives. Clin. Adv. Hematol. Oncol..

[22] Jiricny J. (2006). The multifaceted mismatch-repair system. Nat. Rev. Mol. Cell. Biol..

[23] Des Guetz G., Schischmanoff O., Nicolas P., Perret G.-Y., Morere J.-F., Uzzan B. (2009). Does microsatellite instability predict the efficacy of adjuvant chemotherapy in colorectal cancer? A systematic review with meta-analysis. Eur. J. Cancer.

[24] Guastadisegni C., Colafranceschi M., Ottini L., Dogliotti E. (2010). Microsatellite instability as a marker of prognosis and response to therapy: A meta-analysis of colorectal cancer survival data. Eur. J. Cancer.

[25] Wang Z., Zhao X., Gao C., Gong J., Wang X., Gao J., Li Z., Wang J., Yang B., Wang L. (2020). Plasma-based microsatellite instability detection strategy to guide immune checkpoint blockade treatment. J. Immunother. Cancer.

[26] Sepulveda A.R., Hamilton S.R., Allegra C.J., Grody W., Cushman-Vokoun A.M., Funkhouser W.K., Kopetz S.E., Lieu C., Lindor N.M., Minsky B.D. (2017). Molecular Biomarkers for the Evaluation of Colorectal Cancer: Guideline from the American Society for Clinical Pathology, College of American Pathologists, Association for Molecular Pathology, and the American Society of Clinical Oncology. J. Clin. Oncol..

[27] Gupta S., Provenzale D., Llor X., Halverson A.L., Grady W., Chung D.C., Haraldsdottir S., Markowitz A.J., Jr T.P.S., Hampel H. (2019). NCCN Guidelines Insights: Genetic/Familial High-Risk Assessment: Colorectal, Version 2.2019: Featured Updates to the NCCN Guidelines. J. Natl. Comprehens. Cancer Netw..

[28] Boland C.R., Goel A. (2010). Microsatellite Instability in Colorectal Cancer. Gastroenterology.

[29] Nojadeh J.N., Behrouz Sharif S., Sakhinia E. (2018). Microsatellite instability in colorectal cancer. EXCLI J..

[30] Chen L., Pan X., Hu X., Zhang Y.-H., Wang S., Huang T., Cai Y.-D. (2018). Gene expression differences among different MSI statuses in colorectal cancer. Int. J. Cancer.

[31] Li K., Luo H., Huang L., Luo H., Zhu X. (2020). Microsatellite instability: A review of what the oncologist should know. Cancer Cell Int..

[32] Bonneville R., Krook M.A., Chen H.-Z., Smith A., Samorodnitsky E., Wing M.R., Reeser J.W., Roychowdhury S. (2020). Detection of microsatellite instability biomarkers via next-generation sequencing. Methods Mol. Biol..

[33] Cohen S.J., Punt C.J., lannotti N., Saidman B.H., Sabbath K.D., Gabrail N.Y., Picus J., Morse M., Mitchell E., Miller M.C. (2008). Relationship of Circulating Tumor Cells to Tumor Response, Progression-Free Survival, and Overall Survival in Patients With Metastatic Colorectal Cancer. J. Clin. Oncol..

[34] Vilar E., Gruber S.B. (2010). Microsatellite instability in colorectal cancer-the stable evidence. Nat. Rev. Clin. Oncol..

[35] Tomlinson I., Halford S., Aaltonen L., Hawkins N., Ward R. (2002). Does MSI-low exist?. J. Pathol..

[36] Ogino S., Goel A. (2008). Molecular Classification and Correlates in Colorectal Cancer. J. Mol. Diagn..

[37] Jass J.R. (2007). Classification of colorectal cancer based on correlation of clinical, morphological and molecular features. Histopathology.

[38] Melo F.D.S.E., Wang X., Jansen M., Fessler E., Trinh A., de Rooij L.P.M.H., de Jong J.H., de Boer O.J., van Leersum R., Bijlsma M.F. (2013). Poor-prognosis colon cancer is defined by a molecularly distinct subtype and develops from serrated precursor lesions. Nat. Med..

[39] Marisa L., de Reyniès A., Duval A., Selves J., Gaub M.P., Vescovo L., Etienne-Grimaldi M.-C., Schiappa R., Guenot D., Ayadi M. (2013). Gene Expression Classification of Colon Cancer into Molecular Subtypes: Characterization, Validation, and Prognostic Value. PLoS Med..

[40] Roepman P., Schlicker A., Tabernero J., Majewski I., Tian S., Moreno V., Snel M.H., Chresta C.M., Rosenberg R., Nitsche U. (2014). Colorectal cancer intrinsic subtypes predict chemotherapy benefit, deficient mismatch repair and epithelial-to-mesenchymal transition. Int. J. Cancer.

[41] Singh M.P., Rai S., Pandey A., Singh N.K., Srivastava S. (2019). Molecular subtypes of colorectal cancer: An emerging therapeutic opportunity for personalized medicine. Genes Dis..

[42] Muzny D.M., Bainbridge M.N., Chang K., Dinh H.H., Drummond J.A., Fowler G., Kovar C.L., Lewis L.R., Morgan M.B., Newsham I.F. (2012). Comprehensive molecular characterization of human colon and rectal cancer. Nature.

[43] Guinney J., Dienstmann R., Wang X., de Reyniès A., Schlicker A., Soneson C., Marisa L., Roepman P., Nyamundanda G., Angelino P. (2015). The Consensus Molecular Subtypes of Colorectal Cancer. Nat. Med..

[44] Alderdice M., Richman S.D., Gollins S., Stewart J.P., Hurt C., Adams R., McCorry A.M., Roddy A.C., Vimalachandran D., Isella C. (2018). Prospective patient stratification into robust cancer-cell intrinsic subtypes from colorectal cancer biopsies. J. Pathol..

[45] Sawayama H., Miyamoto Y., Ogawa K., Yoshida N., Baba H. (2020). Investigation of colorectal cancer in accordance with consensus molecular subtype classification. Ann. Gastroenterol. Surg..

[46] Umar A., Boland C.R., Terdiman J.P., Syngal S., de la Chapelle A., Rüschoff J., Fishel R., Lindor N.M., Burgart L.J., Hamelin R. (2004). Revised Bethesda Guidelines for hereditary nonpolyposis colorectal cancer (Lynch syndrome) and microsatellite instability. J. Natl. Cancer Inst..

[47] You J.-F., Buhard O., Ligtenberg M.J.L., Kets C.M., Niessen R.C., Hofstra R.M.W., Wagner A., Dinjens W.N.M., Colas C., Lascols O. (2010). Tumours with loss of MSH6 expression are MSI-H when screened with a pentaplex of five mononucleotide repeats. Br. J. Cancer.

[48] Cicek M.S., Lindor N.M., Gallinger S., Bapat B., Hopper J.L., Jenkins M.A., Young J., Buchanan D., Walsh M.D., Le Marchand L. (2011). Quality assessment and correlation of microsatellite instability and immunohistochemical markers among population- and clinic-based colorectal tumors results from the Colon Cancer Family Registry. J. Mol. Diagn..

[49] Setaffy L., Langner C. (2015). Microsatellite instability in colorectal cancer: Clinicopathological significance. Pol. J. Pathol..

[50] Hu W., Yang Y., Qi L., Chen J., Ge W., Zheng S. (2019). Subtyping of microsatellite instability-high colorectal cancer. Cell Commun. Signal..

[51] Sargent D.J., Marsoni S., Monges G., Thibodeau S.N., Labianca R., Hamilton S.R., French A.J., Kabat B., Foster N.R., Torri V. (2010). Defective mismatch repair as a predictive marker for lack of efficacy of fluorouracil-based adjuvant therapy in colon cancer. J. Clin. Oncol..

[52] Hutchins G., Southward K., Handley K., Magill L., Beaumont C., Stahlschmidt J., Richman S., Chambers P., Seymour M., Kerr D. (2011). Value of mismatch repair, KRAS, and BRAF mutations in predicting recurrence and benefits from chemotherapy in colorectal cancer. J. Clin. Oncol..

[53] Paulose R.R., Ail D.A., Biradar S., Vasudevan A., Sundaram K.R. (2019). Prognostic and predictive significance of microsatellite instability in stage II colorectal carcinoma: An 8-year study from a tertiary center in South India. Ind. J. Cancer.

[54] Joosse S.A., Pantel K. (2016). Genetic traits for hematogeneous tumor cell dissemination in cancer patients. Cancer Metastasis Rev..

[55] Bellizzi A.M., Frankel W.L. (2009). Colorectal cancer due to deficiency in DNA mismatch repair function: A review. Adv. Anat. Pathol..

[56] Goldstein J.B., Wu W., Borras E., Masand G., Cuddy A., Mork M.E., Bannon S.A., Lynch P.M., Rodriguez-Bigas M., Taggart M.W. (2017). Can Microsatellite Status of Colorectal Cancer Be Reliably Assessed after Neoadjuvant Therapy?. Clin. Cancer Res..

[57] Shia J. (2008). Immunohistochemistry versus microsatellite instability testing for screening colorectal cancer patients at risk for hereditary nonpolyposis colorectal cancer syndrome. Part I. The utility of immunohistochemistry. J. Mol. Diagn..

[58] Zhang X., Li J. (2013). Era of universal testing of microsatellite instability in colorectal cancer. World J. Gastrointest. Oncol..

[59] Zhang L. (2008). Immunohistochemistry versus microsatellite instability testing for screening colorectal cancer patients at risk for hereditary nonpolyposis colorectal cancer syndrome. Part II. The utility of microsatellite instability testing. J. Mol. Diagn..

[60] Willis J., Lefterova M.I., Artyomenko A., Kasi P.M., Nakamura Y., Mody K., Catenacci D.V.T., Fakih M., Barbacioru C., Zhao J. (2019). Validation of Microsatellite Instability Detection Using a Comprehensive Plasma-Based Genotyping Panel. Clin. Cancer Res..

[61] Deng A., Yang J., Lang J., Jiang Z., Wang W., Yuan D., Wang X., Tian G. (2018). Monitoring microsatellite instability (MSI) in circulating tumor DNA by next-generation DNA-seq. J. Clin. Oncol..

[62] Mathai R.A., Vidya R.V.S., Reddy B.S., Thomas L., Udupa K., Kolesar J., Rao M. (2019). Potential Utility of Liquid Biopsy as a Diagnostic and Prognostic Tool for the Assessment of Solid Tumors: Implications in the Precision Oncology. J. Clin. Med..

[63] Berretta M., Alessandrini L., De Divitiis C., Nasti G., Lleshi A., Di Francia R., Facchini G., Cavaliere C., Buonerba C., Canzonieri V. (2017). Serum and tissue markers in colorectal cancer: State of art. Crit. Rev. Oncol. Hematol..

[64] Huang Z., Huang D., Ni S., Peng Z., Sheng W., Du X. (2010). Plasma microRNAs are promising novel biomarkers for early detection of colorectal cancer. Int. J. Cancer.

[65] Liu Z., Zhang Y., Niu Y., Li K., Liu X., Chen H., Gao C. (2014). A Systematic Review and Meta-Analysis of Diagnostic and Prognostic Serum Biomarkers of Colorectal Cancer. PLoS ONE.

[66] Bhardwaj M., Gies A., Werner S., Schrotz-King P., Brenner H. (2017). Blood-Based Protein Signatures for Early Detection of Colorectal Cancer: A Systematic Review. Clin. Transl. Gastroenterol..

[67] Vatandoost N., Ghanbari J., Mojaver M., Avan A., Ghayour-Mobarhan M., Nedaeinia R., Salehi R. (2016). Early detection of colorectal cancer: From conventional methods to novel biomarkers. J. Cancer Res. Clin. Oncol..

[68] Kuppusamy P., Govindan N., Yusoff M.M., Ichwan S.J.A. (2017). Proteins are potent biomarkers to detect colon cancer progression. Saudi J. Biol. Sci..

[69] Ahn S.B., Sharma S., Mohamedali A., Mahboob S., Redmond W.J., Pascovici D., Wu J.X., Zaw T., Adhikari S., Vaibhav V. (2019). Potential early clinical stage colorectal cancer diagnosis using a proteomics blood test panel. Clin. Proteom..

[70] Borrebaeck C.A.K. (2017). Precision diagnostics: Moving towards protein biomarker signatures of clinical utility in cancer. Nat. Rev. Cancer.

[71] Tieng F.Y.F., Abu N., Sukor S., Mohd Azman Z.A., Mahamad Nadzir N., Lee L.-H., Ab Mutalib N.S. (2020). L1CAM, CA9, KLK6, HPN, and ALDH1A1 as Potential Serum Markers in Primary and Metastatic Colorectal Cancer Screening. Diagnostics.

[72] Berger B.M., Ahlquist D.A. (2012). Stool DNA screening for colorectal neoplasia: Biological and technical basis for high detection rates. Pathology.

[73] Liu R., Su X., Long Y., Zhou D., Zhang X., Ye Z., Ma J., Tang T., Wang F., He C. (2019). A systematic review and quantitative assessment of methylation biomarkers in fecal DNA and colorectal cancer and its precursor, colorectal adenoma. Mutat. Res..

[74] Worm Ørntoft M.-B. (2018). Review of Blood-Based Colorectal Cancer Screening: How Far Are Circulating Cell-Free DNA Methylation Markers From Clinical Implementation?. Clin. Colorectal Cancer.

[75] Rasmussen S.L., Krarup H.B., Sunesen K.G., Johansen M.B., Stender M.T., Pedersen I.S., Madsen P.H., Thorlacius-Ussing O. (2017). Hypermethylated DNA, a circulating biomarker for colorectal cancer detection. PLoS ONE.

[76] Ab Mutalib N.-S., Md Yusof N.F., Abdul S.-N., Jamal R. (2017). Pharmacogenomics DNA Biomarkers in Colorectal Cancer: Current Update. Front. Pharmacol..

[77] Ab Mutalib N.-S., Baharuddin R., Jamal R. (2019). Epigenome-Wide Analysis of DNA Methylation in Colorectal Cancer. Computational Epigenetics and Diseases.

[78] Luo X., Burwinkel B., Tao S., Brenner H. (2011). MicroRNA Signatures: Novel Biomarker for Colorectal Cancer?. Cancer Epidemiol. Biomark. Prev..

[79] Abedini P., Fattahi A., Agah S., Talebi A., Beygi A.H., Amini S.M., Mirzaei A., Akbari A. (2019). Expression analysis of circulating plasma long noncoding RNAs in colorectal cancer: The relevance of lncRNAs ATB and CCAT1 as potential clinical hallmarks. J. Cell. Physiol..

[80] Chen B., Xia Z., Deng Y.-N., Yang Y., Zhang P., Zhu H., Xu N., Liang S. (2019). Emerging microRNA biomarkers for colorectal cancer diagnosis and prognosis. Open Biol..

[81] Bastaminejad S., Taherikalani M., Ghanbari R., Akbari A., Shabab N., Saidijam M. (2017). Investigation of MicroRNA-21 Expression Levels in Serum and Stool as a Potential Non-Invasive Biomarker for Diagnosis of Colorectal Cancer. Iran. Biomed. J..

[82] Mohd Yunos R.I., Ab Mutalib N.S., Tieng F.Y.F., Abu N., Jamal R. (2020). Actionable Potentials of Less Frequently Mutated Genes in Colorectal Cancer and Their Roles in Precision Medicine. Biomolecules.

[83] Baharudin R., Tieng F.Y.F., Lee L.-H., Ab Mutalib N.S. (2020). Epigenetics of SFRP1: The Dual Roles in Human Cancers. Cancers.

[84] Loktionov A. (2020). Biomarkers for detecting colorectal cancer non-invasively: DNA, RNA or proteins?. World J. Gastrointest. Oncol..

[85] Norcic G. (2018). Liquid Biopsy in Colorectal Cancer-Current Status and Potential Clinical Applications. Micromachines.

[86] Odegaard J.I., Vincent J.J., Mortimer S., Vowles J.V., Ulrich B.C., Banks K.C., Fairclough S.R., Zill O.A., Sikora M., Mokhtari R. (2018). Validation of a Plasma-Based Comprehensive Cancer Genotyping Assay Utilizing Orthogonal Tissue- and Plasma-Based Methodologies. Clin. Cancer Res..

[87] Kolenčík D., Shishido S.N., Pitule P., Mason J., Hicks J., Kuhn P. (2020). Liquid Biopsy in Colorectal Carcinoma: Clinical Applications and Challenges. Cancers.

[88] Kopetz S., Lefterova M. Microsatellite Instability Detection Via Liquid Biopsy Test Shows High Concordance With Results From Tissue Samples. https://www.aacr.org:443/Newsroom/Pages/News-Release-Detail.aspx?ItemID=1328.

[89] Silveira A.B., Bidard F.-C., Kasperek A., Melaabi S., Tanguy M.-L., Rodrigues M., Bataillon G., Cabel L., Buecher B., Pierga J.-Y. (2020). High-Accuracy Determination of Microsatellite Instability Compatible with Liquid Biopsies. Clin. Chem..

[90] Gargalionis A.N., Papavassiliou A.G. (2017). Liquid Biopsies in Colorectal Cancer: Monitoring Genetic Heterogeneity. Trends Cancer.

[91] Makrigiorgos G., Ladas I., Mamon H.J., Ng K., Yu F., Leong C.K., Kulke M. (2019). Sensitive Detection of Microsatellite Instability (MSI) in Liquid Biopsies from Early Stage Colon Cancer Patients using Nuclease-based Enrichment and Standard-Marker or NGS based approaches. Int. J. Radiat. Oncol. Biol. Phys..

[92] Baudrin L.G., Deleuze J.-F., How-Kit A. (2018). Molecular and Computational Methods for the Detection of Microsatellite Instability in Cancer. Front. Oncol..

[93] Cortes-Ciriano I., Lee S., Park W.-Y., Kim T.-M., Park P.J. (2017). A molecular portrait of microsatellite instability across multiple cancers. Nat. Commun..

[94] Alix-Panabières C., Pantel K. (2016). Clinical Applications of Circulating Tumor Cells and Circulating Tumor DNA as Liquid Biopsy. Cancer Discov..

[95] Merker J.D., Oxnard G.R., Compton C., Diehn M., Hurley P., Lazar A.J., Lindeman N., Lockwood C.M., Rai A.J., Schilsky R.L. (2018). Circulating Tumor DNA Analysis in Patients with Cancer: American Society of Clinical Oncology and College of American Pathologists Joint Review. J. Clin. Oncol..

[96] Stewart C.M., Kothari P.D., Mouliere F., Mair R., Somnay S., Benayed R., Zehir A., Weigelt B., Dawson S.-J., Arcila M.E. (2018). The value of cell-free DNA for molecular pathology. J. Pathol..

[97] Fiala C., Diamandis E.P. (2019). New approaches for detecting cancer with circulating cell-free DNA. BMC Med..

[98] Bi F., Wang Q., Dong Q., Wang Y., Zhang L., Zhang J. (2020). Circulating tumor DNA in colorectal cancer: Opportunities and challenges. Am. J. Transl. Res..

[99] Heitzer E., Haque I.S., Roberts C.E.S., Speicher M.R. (2019). Current and future perspectives of liquid biopsies in genomics-driven oncology. Nat. Rev. Genet..

[100] Haque I.S., Elemento O. (2017). Challenges in Using ctDNA to Achieve Early Detection of Cancer. bioRxiv.

[101] Bedin C., Enzo M.V., Del Bianco P., Pucciarelli S., Nitti D., Agostini M. (2017). Diagnostic and prognostic role of cell-free DNA testing for colorectal cancer patients. Int. J. Cancer.

[102] Osumi H., Shinozaki E., Yamaguchi K., Zembutsu H. (2019). Clinical utility of circulating tumor DNA for colorectal cancer. Cancer Sci..

[103] Antoniotti C., Pietrantonio F., Corallo S., De Braud F., Falcone A., Cremolini C. (2019). Circulating Tumor DNA Analysis in Colorectal Cancer: From Dream to Reality. J. Clin. Oncol. Precis. Oncol..

[104] Calapre L., Warburton L., Millward M., Gray E.S. (2019). Circulating tumour DNA (ctDNA) as a biomarker in metachronous melanoma and colorectal cancer—A case report. BMC Cancer.

[105] Kasi P.M. (2017). Mutational burden on circulating cell-free tumor-DNA testing as a surrogate marker of mismatch repair deficiency or microsatellite instability in patients with colorectal cancers. J. Gastrointest. Oncol..

[106] Barzi A., Campan M., Petterson J., Du L., Long T., Dubeau L., Lenz H.-J., Ward P. (2018). Assessment of microsatellite instability (MSI) in cell free DNA (cfDNA) of colorectal cancers (CRC) patients (pts). J. Clin. Oncol..

[107] Georgiadis A., Durham J.N., Keefer L.A., Bartlett B.R., Zielonka M., Murphy D., White J.R., Lu S., Verner E.L., Ruan F. (2019). Noninvasive Detection of Microsatellite Instability and High Tumor Mutation Burden in Cancer Patients Treated with PD-1 Blockade. Clin. Cancer Res..

[108] Wang L., Ajani J.A. (2019). Ushering in Liquid Biopsy for the Microsatellite Status: Advantages and Caveats. Clin. Cancer Res..

[109] Isaacs J., Nixon A.B., Bolch E., Quinn K., Banks K., Hanks B.A., Strickler J.H. (2019). Blood-based genomic profiling of cell-free DNA (cfDNA) to identify microsatellite instability (MSI-H), tumor mutational burden (TMB) and Wnt/B-Catenin pathway alterations in patients with gastrointestinal (GI) tract cancers. J. Clin. Oncol..

[110] Hindson B.J., Ness K.D., Masquelier D.A., Belgrader P., Heredia N.J., Makarewicz A.J., Bright I.J., Lucero M.Y., Hiddessen A.L., Legler T.C. (2011). High-throughput droplet digital PCR system for absolute quantitation of DNA copy number. Anal. Chem..

[111] Taylor S.C., Laperriere G., Germain H. (2017). Droplet Digital PCR versus qPCR for gene expression analysis with low abundant targets: From variable nonsense to publication quality data. Sci. Rep..

[112] Lee H.S., Kim W.H., Kwak Y., Koh J., Bae J.M., Kim K.-M., Chang M.S., Han H.S., Kim J.M., Kim H.W. (2017). Molecular Testing for Gastrointestinal Cancer. J. Pathol. Transl. Med..

[113] Ladas I., Yu F., Leong K.W., Fitarelli-Kiehl M., Song C., Ashtaputre R., Kulke M., Mamon H., Makrigiorgos G.M. (2018). Enhanced detection of microsatellite instability using pre-PCR elimination of wild-type DNA homo-polymers in tissue and liquid biopsies. Nucleic Acids Res..

[114] Evrard C., Tachon G., Randrian V., Karayan-Tapon L., Tougeron D. (2019). Microsatellite Instability: Diagnosis, Heterogeneity, Discordance, and Clinical Impact in Colorectal Cancer. Cancers.

[115] Allen-Mersh T.G., McCullough T.K., Patel H., Wharton R.Q., Glover C., Jonas S.K. (2007). Role of circulating tumour cells in predicting recurrence after excision of primary colorectal carcinoma. Br. J. Surg..

[116] Burz C., Pop V.-V., Buiga R., Daniel S., Samasca G., Aldea C., Lupan I. (2018). Circulating tumor cells in clinical research and monitoring patients with colorectal cancer. Oncotarget.

[117] Mohamed Suhaimi N.-A., Foong Y.M., Lee D.Y.S., Phyo W.M., Cima I., Lee E.X.W., Goh W.L., Lim W.-Y., Chia K.S., Kong S.L. (2015). Non-invasive sensitive detection of KRAS and BRAF mutation in circulating tumor cells of colorectal cancer patients. Mol. Oncol..

[118] Tellez-Gabriel M., Heymann M.-F., Heymann D. (2019). Circulating Tumor Cells as a Tool for Assessing Tumor Heterogeneity. Theranostics.

[119] Toh J.W.T., Lim S.H., MacKenzie S., de Souza P., Bokey L., Chapuis P., Spring K.J. (2020). Association between Microsatellite Instability Status and Peri-Operative Release of Circulating Tumour Cells in Colorectal Cancer. Cells.

[120] Tan C.R.C., Zhou L., El-Deiry W.S. (2016). Circulating Tumor Cells Versus Circulating Tumor DNA in Colorectal Cancer: Pros and Cons. Curr. Colorectal Cancer Rep..

[121] Steinert G., Schölch S., Niemietz T., Iwata N., García S.A., Behrens B., Voigt A., Kloor M., Benner A., Bork U. (2014). Immune Escape and Survival Mechanisms in Circulating Tumor Cells of Colorectal Cancer. Cancer Res..

[122] Kong S.L., Liu X., Suhaimi N.-A.M., Koh K.J.H., Hu M., Lee D.Y.S., Cima I., Phyo W.M., Lee E.X.W., Tai J.A. (2017). Molecular characterization of circulating colorectal tumor cells defines genetic signatures for individualized cancer care. Oncotarget.

[123] Messaritakis I., Sfakianaki M., Vogiatzoglou K., Koulouridi A., Dimitriou O., Gouvas N., Athanasakis E., Tsiaoussis I., Xynos E., Mavroudis D. (2019). P-079Circulating tumor cell detection and microsatellite instability status in predicting outcomes of advanced CRC patients. Ann. Oncol..

[124] Han X., Zhang S., Zhou D.C., Wang D., He X., Yuan D., Li R., He J., Duan X., Wendl M.C. (2021). MSIsensor-ct: Microsatellite instability detection using cfDNA sequencing data. Brief. Bioinform..

[125] Georgiadis A., Sausen M. (2018). Process for Microsatellite Instability. Detection. Patent.

[126] Huang X. (2018). MSI from Liquid Biopsies. WO Patent.

[127] Rabizadeh S. (2019). Assessing Microsatellite Instability by Liquid Biopsy. WO Patent.

[128] Detection of MSI in Circulating Tumor DNA of Colorectal Carcinoma Patients—Full Text View—ClinicalTrials.gov. https://clinicaltrials.gov/ct2/show/NCT03594448.

[129] Detect Microsatellite Instability Status in Blood Sample of Advanced Colorectal Cancer Patients by Next-Generation Sequencing—Full Text View—ClinicalTrials.gov. https://clinicaltrials.gov/ct2/show/NCT03561350.

[130] Study of Pembrolizumab (MK-3475) vs Standard Therapy in Participants with Microsatellite Instability-High (MSI-H) or Mismatch Repair Deficient (dMMR) Stage IV Colorectal Carcinoma (MK-3475-177/KEYNOTE-177)—Full Text View—ClinicalTrials.gov. https://clinicaltrials.gov/ct2/show/NCT02563002.

[131] Le D.T., Kim T.W., Van Cutsem E., Geva R., Jäger D., Hara H., Burge M., O’Neil B., Kavan P., Yoshino T. (2019). Phase II Open-Label Study of Pembrolizumab in Treatment-Refractory, Microsatellite Instability–High/Mismatch Repair–Deficient Metastatic Colorectal Cancer: KEYNOTE-164. J. Clin. Oncol..

[132] Bai Y., Zhao H. (2018). Liquid biopsy in tumors: Opportunities and challenges. Ann. Transl. Med..

[133] Carroll M.R.R., Seaman H.E., Halloran S.P. (2014). Tests and investigations for colorectal cancer screening. Clin. Biochem..

[134] Pox C.P., Altenhofen L., Brenner H., Theilmeier A., Stillfried D.V., Schmiegel W. (2012). Efficacy of a Nationwide Screening Colonoscopy Program for Colorectal Cancer. Gastroenterology.

[135] Brenner H., Hoffmeister M., Arndt V., Stegmaier C., Altenhofen L., Haug U. (2010). Protection From Right- and Left-Sided Colorectal Neoplasms After Colonoscopy: Population-Based Study. J. Natl. Cancer Inst..

[136] Morikawa T., Kato J., Yamaji Y., Wada R., Mitsushima T., Shiratori Y. (2005). A Comparison of the Immunochemical Fecal Occult Blood Test and Total Colonoscopy in the Asymptomatic Population. Gastroenterology.

[137] Haug U., Hundt S., Brenner H. (2010). Quantitative Immunochemical Fecal Occult Blood Testing for Colorectal Adenoma Detection: Evaluation in the Target Population of Screening and Comparison with Qualitative Tests. Am. J. Gastroenterol..

[138] Quandt D., Zucht H.D., Amann A., Wulf-Goldenberg A., Borrebaeck C., Cannarile M., Lambrechts D., Oberacher H., Garrett J., Nayak T. (2017). Implementing liquid biopsies into clinical decision making for cancer immunotherapy. Oncotarget.

[139] Keller L., Werner S., Pantel K. (2019). Biology and clinical relevance of EpCAM. Cell Stress.

[140] Belloum Y., Janning M., Mohme M., Simon R., Kropidlowski J., Sartori A., Irwin D., Westphal M., Lamszus K., Loges S. (2020). Discovery of Targetable Genetic Alterations in NSCLC Patients with Different Metastatic Patterns Using a MassARRAY-Based Circulating Tumor DNA Assay. Cells.

[141] Cescon D.W., Bratman S.V., Chan S.M., Siu L.L. (2020). Circulating tumor DNA and liquid biopsy in oncology. Nat. Cancer.

[142] Ignatiadis M., Sledge G.W., Jeffrey S.S. (2021). Liquid biopsy enters the clinic—Implementation issues and future challenges. Nat. Rev. Clin. Oncol..

[143] Fleischhacker M., Schmidt B. (2020). Pre-analytical issues in liquid biopsy—Where do we stand?. J. Lab. Med..

[144] Salvianti F., Gelmini S., Costanza F., Mancini I., Sonnati G., Simi L., Pazzagli M., Pinzani P. (2020). The pre-analytical phase of the liquid biopsy. New Biotechnol..

[145] Neumann M.H.D., Bender S., Krahn T., Schlange T. (2018). ctDNA and CTCs in Liquid Biopsy—Current Status and Where We Need to Progress. Comput. Struct. Biotechnol. J..

[146] Ding Y., Li W., Wang K., Xu C., Hao M., Ding L. (2020). Perspectives of the Application of Liquid Biopsy in Colorectal Cancer. BioMed Res..

[147] Siravegna G., Marsoni S., Siena S., Bardelli A. (2017). Integrating liquid biopsies into the management of cancer. Nat. Rev. Clin. Oncol..

[148] Talseth-Palmer B.A., Bauer D.C., Sjursen W., Evans T.J., McPhillips M., Proietto A., Otton G., Spigelman A.D., Scott R.J. (2016). Targeted next-generation sequencing of 22 mismatch repair genes identifies Lynch syndrome families. Cancer Med..

[149] Bettegowda C., Sausen M., Leary R.J., Kinde I., Wang Y., Agrawal N., Bartlett B.R., Wang H., Luber B., Alani R.M. (2014). Detection of Circulating Tumor DNA in Early- and Late-Stage Human Malignancies. Sci. Transl. Med..

[150] Castro-Giner F., Gkountela S., Donato C., Alborelli I., Quagliata L., Ng C.K.Y., Piscuoglio S., Aceto N. (2018). Cancer Diagnosis Using a Liquid Biopsy: Challenges and Expectations. Diagnostics.

[151] Vanderwalde A., Spetzler D., Xiao N., Gatalica Z., Marshall J. (2018). Microsatellite instability status determined by next-generation sequencing and compared with PD-L1 and tumor mutational burden in 11,348 patients. Cancer Med..

[152] Chen L., Liu P., Evans T.C., Ettwiller L.M. (2017). DNA damage is a pervasive cause of sequencing errors, directly confounding variant identification. Science.

[153] Costello M., Pugh T.J., Fennell T.J., Stewart C., Lichtenstein L., Meldrim J.C., Fostel J.L., Friedrich D.C., Perrin D., Dionne D. (2013). Discovery and characterization of artifactual mutations in deep coverage targeted capture sequencing data due to oxidative DNA damage during sample preparation. Nucleic Acids Res..

[154] Pécuchet N., Rozenholc Y., Zonta E., Pietrasz D., Didelot A., Combe P., Gibault L., Bachet J.-B., Taly V., Fabre E. (2016). Analysis of Base-Position Error Rate of Next-Generation Sequencing to Detect Tumor Mutations in Circulating DNA. Clin. Chem..

[155] Lim S.B., Lim C.T., Lim W.-T. (2019). Single-Cell Analysis of Circulating Tumor Cells: Why Heterogeneity Matters. Cancers.

[156] Zhou Y., Wang C., Zhu C., Chen J., Cheng M., Deng Y., Guo Y. (2017). Single-cell gene variation analysis method for single gland. Hereditas.

[157] Lim S.B., Di Lee W., Vasudevan J., Lim W.-T., Lim C.T. (2019). Liquid biopsy: One cell at a time. NPJ Precis. Oncol..

[158] Tieng F.Y.F., Baharudin R., Abu N., Mohd Yunos R.-I., Lee L.-H., Ab Mutalib N.-S. (2020). Single Cell Transcriptome in Colorectal Cancer—Current Updates on Its Application in Metastasis, Chemoresistance and the Roles of Circulating Tumor Cells. Front. Pharmacol..

